# Baicalin Inhibits NOD-Like Receptor Family, Pyrin Containing Domain 3 Inflammasome Activation in Murine Macrophages by Augmenting Protein Kinase A Signaling

**DOI:** 10.3389/fimmu.2017.01409

**Published:** 2017-10-27

**Authors:** Chen-Guang Li, Liang Yan, Feng-Yi Mai, Zi-Jian Shi, Li-Hui Xu, Yan-Yun Jing, Qing-Bing Zha, Dong-Yun Ouyang, Xian-Hui He

**Affiliations:** ^1^Department of Immunobiology, College of Life Science and Technology, Jinan University, Guangzhou, China; ^2^Department of Fetal Medicine, The First Affiliated Hospital of Jinan University, Guangzhou, China; ^3^Department of Cell Biology, College of Life Science and Technology, Jinan University, Guangzhou, China

**Keywords:** baicalin, NOD-like receptor (NLR) family, pyrin containing domain 3 inflammasome, interleukin-1β, protein kinase A, macrophages

## Abstract

The flavonoid baicalin has been reported to possess potent anti-inflammatory activities by suppressing inflammatory signaling pathways. However, whether baicalin can suppress the activation of NOD-like receptor (NLR) family, pyrin containing domain 3 (NLRP3) inflammasome in macrophages is largely unknown. Here, we showed that baicalin treatment dose-dependently inhibited adenosine triphosphate (ATP) or nigericin-induced NLRP3 inflammasome activation, as revealed by the decreased release of mature interleukin (IL)-1β, active caspase-1p10, and high-mobility group box-1 protein from lipopolysaccharide (LPS)-primed bone marrow-derived macrophages. The formation of ASC specks, a critical marker of NLRP3 inflammasome assembly, was robustly inhibited by baicalin in the macrophages upon ATP or nigericin stimulation. All these inhibitory effects of baicalin could be partly reversed by MDL12330A or H89, both of which are inhibitors of the protein kinase A (PKA) signaling pathway. Consistent with this, baicalin strongly enhanced PKA-mediated phosphorylation of NLRP3, which has been suggested to prevent ASC recruitment into the inflammasome. Of note, the PKA inhibitor H89 could block baicalin-induced NLRP3 phosphorylation on PKA-specific sites, further supporting PKA’s role in this process. In addition, we showed that when administered pre and post exposure to *Escherichia coli* infection baicalin treatment significantly improved mouse survival in bacterial sepsis. Baicalin administration also significantly reduced IL-1β levels in the sera of bacterial infected mice. Altogether, our results revealed that baicalin inhibited NLRP3 inflammasome activation at least partly through augmenting PKA signaling, highlighting its therapeutic potential for the treatment of NLRP3-related inflammatory diseases.

## Introduction

The NOD-like receptor (NLR) family, pyrin containing domain 3 (NLRP3) is an intracellular sensing protein that can be activated by diverse factors from pathogens, environments, and hosts (1, 2). When pathogen-associated molecular patterns (PAMPs) are bound to and recognized by the pattern recognition receptors, the nuclear factor (NF)-κB pathway is activated leading to the expression of pro-interleukin (IL)-1β, pro-IL-18, and NLRP3 (2, 3). NLRP3 can be further triggered by a variety of danger-associated molecular patterns (DAMPs), including adenosine triphosphate (ATP) derived from bacterial and host cells (4–6). Subsequently, NLRP3 recruits the adaptor molecule ASC (apoptosis-associated speck-like protein containing a caspase recruitment domain) which in turn interacts with pro-caspase-1 to form a large multi-protein complex called inflammasome. This culminates in the activation of caspase-1, thus converting pro-IL-1β and pro-IL-18 to their mature forms (1–3). Concomitantly, the active caspase-1 also cleaves gasdermin D protein to release its active N-terminal fragment, the latter of which forms pores in the plasma membrane leading to an inflammatory form of cell death named pyroptosis (7–13). Interestingly, recent studies indicated that the release of mature IL-1β is dependent on pyroptosis in macrophages (7, 11). As released IL-1β and high-mobility group box-1 (HMGB1) can further intensify the innate immunity, NLRP3 inflammasome activation constitutes a first line of defense against pathogenic infections (14, 15).

Although NLRP3 inflammasome has critical roles in combating pathogens, excessive or constitutive activation of NLRP3 inflammasome has been implicated in many inflammatory diseases in the contexts of infections, sterile tissue damages, and metabolic dysfunctions (16, 17). This is partially due to the excessive secretion of inflammatory cytokines like IL-1β upon NLRP3 inflammasome activation (18). These cytokines in turn enhance pyroptosis leading to multiple organ damage and septic death during bacterial infections (19, 20). Consistent with this notion, hyperactive NLRP3 due to conditional NLRP3 mutant knock-in resulted in hepatocyte pyroptosis, liver injury, and shortened survival of the experimental mice (19). On the contrary, blocking pyroptosis by *caspase-1/-11* or *gasdermin D* gene deletion confers the mice resistance to endotoxin-induced sepsis (20, 21). Furthermore, emerging evidence indicates that NLRP3 hyperactivation contributes to diseases of the central nervous system and lungs (22). NLRP3 inflammasome has also been implicated in the pathogenesis of metabolic disorders such as type 2 diabetes, obesity, atherosclerosis, and gout (16). In addition, the gain-of-function mutations in NLRP3 have been identified as the cause of the inherited cryopyrin-associated periodic syndrome Muckle–Wells syndrome, familial cold autoinflammatory syndrome, and neonatal-onset multisystem inflammatory disease (16, 23). Therefore, controlling NLRP3 inflammasome activation is a promising therapy for the treatment of inflammatory diseases such as bacterial infections, neurological disorders, and metabolic disorders (16).

Baicalin is a flavonoid isolated from the root of *Scutellaria baicalensis* Georgi, a well-known Chinese medicinal plant used to treat fevers (24). It has been demonstrated that baicalin possesses many bioactivities and pharmacological effects, including hepatoprotective, antioxidant, antibacterial, antiviral, and anti-inflammatory activities (24–26). Over past decades, the anti-inflammatory effects of baicalin have been intensively investigated. An early study showed that baicalin could markedly inhibit carrageenan-induced rat paw edema (27). It has also been shown to improve survival in murine model of polymicrobial sepsis induced by cecal ligation and puncture (CLP) (28–30) as well as in the model of endotoxemic shock (31). Several studies have indicated that the antiseptic and anti-inflammatory effects of baicalin are due to inhibition of inflammatory responses *via* downregulating the NF-κB signaling (31–34). There is evidence showing that baicalin may attenuate CLP-induced sepsis by inhibiting the release of HMGB1 and other cytokines including IL-1β (29). Recently, baicalin has been shown to suppress the expression of NLRP3 in LPS-stimulated piglet mononuclear phagocytes by suppressing the NF-κB pathway (35, 36).

Although those studies have revealed that baicalin exhibits potent anti-inflammatory activity likely through inhibiting NF-κB signaling, it is still elusive whether baicalin can affect NLRP3 inflammasome activation by canonical activators including ATP and nigericin. In this study, we found that baicalin robustly suppressed NLRP3 inflammasome activation in LPS-primed macrophages upon ATP or nigericin stimulation. Mechanistically, baicalin blocked ASC recruitment and speck formation in part by augmenting protein kinase A (PKA)-mediated phosphorylation of NLRP3, which has been reported to prevent NLRP3 inflammasome assembly (37, 38). Our results highlight baicalin as an agent for the treatment of NLRP3-related inflammatory diseases by promoting PKA signaling.

## Materials and Methods

### Reagents and Antibodies

Baicalin (572667), MDL12330A (M182), disuccinimidyl suberate (S1885), Hoechst 33342 (B2261), propidium iodide (PI) (P4170), ATP (A6419), lipopolysaccharide (LPS) (*Escherichia coli* O111:B4) (L4391), dimethyl sulfoxide (DMSO) (D8418), Tween-80 (P8074), and Tween-20 (P1379) were bought from Sigma-Aldrich (St. Louis, MO, USA). Baicalin was dissolved in DMSO at 100 mM and stored at −20°C. H89 (S1643), cell lysis buffer for Western and IP (P0013), and phenylmethanesulfonyl fluoride (PMSF) (ST505) were obtained from Beyotime (Haimen, China). Nigericin (#tlrl-nig) was purchased from InvivoGen (San Diego, CA, USA). Dulbecco’s Modified Eagle’s Medium (DMEM) medium with high glucose, fetal bovine serum (FBS), streptomycin, and penicillin, Opti-MEM were products of Thermo Fisher/Gibco (Carlsbad, CA, USA). The anti-NLRP3 antibody (AG-20B-0014) was purchased from Adipogen AG (Liestal, Switzerland). The antibody against caspase-1p10 (M-20) (sc-514) was purchased from Santa Cruz Biotechnology (Dallas, TX, USA). The antibodies against phospho-(Ser/Thr) PKA substrate (#9621), IL-1β (#12242), ASC (#67824), HMGB1 (#3935), β-tubulin (#2128), and horse-radish peroxidase (HRP)-linked horse anti-mouse IgG (#7076), HRP-linked goat anti-rabbit IgG (#7074), and protein G agarose beads (#37478) were purchased from Cell Signaling Technology (Danvers, MA, USA). CF568 goat-anti-rabbit IgG (H + L), highly cross-adsorbed (#20103) and CF488A-conjugated goat-anti-mouse IgG, and highly cross-adsorbed (#20018) were obtained from Biotium (Hayward, CA, USA).

### Experimental Animals

Female C57BL/6 mice (6–8 weeks of age) were bought from the Experimental Animal Center of Southern Medical University (Guangzhou, China). All animals were acclimatized for 1 week before experiments under 12 h dark/12 h light cycle condition. Animal experiments were performed according to the guidelines for the care and use of animals approved by the Committee on the Ethics of Animal Experiments of Jinan University.

### Bone Marrow-Derived Macrophage (BMDM) Culture

Mouse BMDMs were differentiated as reported previously (21, 39). Briefly, mice were sacrificed and bone marrows were collected from the femurs. Bone marrow cells were re-suspended in BM-Mac medium (80% DMEM medium containing 10% FBS plus 20% M-CSF-conditioned medium from L929 cells). Subsequently cells were seeded in 10-cm Petri dish with 10 ml BM-Mac medium and cultured at 37°C in a humidified incubator of 5% CO_2_. BMDMs were ready for experiments after 6 days.

### Cell Death Assay

Cell death was measured by PI incorporation as described previously (39, 40). Cells were cultured in 24-well plates and primed with 500 ng/ml LPS in Opti-MEM for 4 h. Subsequently, cells were treated with indicated concentrations of baicalin in Opti-MEM for 1 h followed by stimulation with ATP (3 mM) for 30 min or nigericin (10 µM) for 1 h. The cells were stained with PI solution (2 µg/ml PI plus 5 µg/ml Hoechst 33342) for 10 min at room temperature and observed immediately by live imaging using Zeiss Axio Observer D1 microscope equipped with a Zeiss LD Plan-Neofluar 20×/0.4 Korr M27 objective lens (Carl Zeiss MicroImaging GmbH, Göttingen, Germany). Fluorescence images were captured with a Zeiss AxioCam MR R3 cooled CCD camera controlled with ZEN software (Carl Zeiss).

### Fluorescence Microscopy

Immunofluorescence analysis was performed as previously described (41, 42). In brief, BMDMs were seeded in glass-bottomed dishes (5 × 10^5^ cells/dish) and cultured at 37°C overnight. Cells were primed with 500 ng/ml LPS in Opti-MEM for 4 h. Then the cells were treated with baicalin for 1 h, followed by treatment with ATP (3 mM) for 30 min or nigericin (10 µM) for 1 h in Opti-MEM. After fixation, permeabilization and blocking, cells were incubated with rabbit anti-ASC antibody (1:300) and mouse anti-NLRP3 antibody (1:300), followed by staining with CF568-conjugated goat-anti-rabbit IgG and CF488A-conjugated goat-anti-mouse IgG. After staining with Hoechst 33342 solution (5 µg/ml in PBS) to reveal the nuclei, the cells were observed under a Zeiss Axio Observer D1 microscope with a Zeiss LD Plan-Neofluar 40×/0.6 Korr M27 objective (Carl Zeiss MicroImaging GmbH, Göttingen, Germany). Fluorescence images were captured by a Zeiss AxioCam MR R3 cooled CCD camera controlled with ZEN software (Carl Zeiss).

### ASC Oligomer Cross-linking

The cross-linking of ASC oligomers was performed as previously described (43, 44). In brief, cells were seeded in 6-well plates at 1.0 × 10^6^ cells/well. After indicated treatments, cells were lysed with cold PBS containing 0.5% Triton-X 100, and the cell lysates were centrifuged at 6,000 × *g* for 15 min at 4°C. The pellets were washed twice with PBS and then re-suspended in 200 µl PBS. 2 mM disuccinimidyl suberate was added to the resuspended pellets, and the suspension was incubated at room temperature for 30 min with rotation. The cross-linked pellets were spun down at 6,000 × *g* for 15 min at 4°C and redissolved in 20 µl of 1× sodium dodecyl sulfate-polyacrylamide gel electrophoresis (SDS-PAGE) sample loading buffer. Samples were boiled for 5 min and analyzed by Western blotting.

### Immunoprecipitation

Cells were rinsed once with ice-cold PBS and lyzed with 0.5 ml ice-cold cell lysis buffer for Western blot and IP (containing 1 mM PMSF). The cell lysates were centrifuged at 13,000 × *g* for 10 min at 4°C, precleared with a 10% volume of Protein G agarose beads for 30 min at 4°C with gentle agitation, and then incubated with anti-NLRP3 antibody (0.5 µg antibody for 100 µg cell lysate) overnight at 4°C with gentle shaking. The antibody-NLRP3 complexes were collected with a 10% volume of Protein G agarose beads for 2 h at 4°C with gentle shaking. The beads were washed five times with cell lysis buffer, boiled for 5 min in 3× SDS-PAGE sample loading buffer, and resolved by Western blot analysis.

### Detection of Soluble IL-1β

Soluble IL-1β in culture supernatants and sera was determined by cytometric bead array (CBA) mouse IL-1β Flex Set (BD Biosciences, San Jose, CA, USA) according to the manufacturer’s instructions. Data were acquired on a flow cytometer (FACSCalibur; Becton Dickinson, Mountain View, CA, USA) equipped with CELLQuest software (Becton Dickinson).

### Precipitation of Soluble Proteins in Supernatants

Soluble protein in culture supernatants was precipitated as previously described (21, 39). The precipitated proteins were dissolved in equal volume of 1× SDS-PAGE sample loading buffer and subjected to Western blot analysis of secreted mature IL-1β, caspase-1p10, and HMGB1.

### Western Blot Analysis

Western blotting was performed essentially as previously described (39). Briefly, total proteins were separated by SDS-PAGE and electro-transferred to PVDF membranes (#03010040001; Roche Diagnostics GmbH, Mannheim, Germany). The membranes were blocked by blocking buffer (PBS containing 3% FBS and 0.1% Tween-20) for 1 h and incubated with indicated primary antibody overnight at 4°C, followed by incubation with appropriate HRP-linked secondary antibody (horse anti-mouse or goat anti-rabbit IgG). Bands were revealed with an enhanced chemiluminescence kit (BeyoECL Plus; Beyotime, Haimen, China) and recorded by X-ray films (Carestream, Xiamen, China). The blot images were captured by FluorChem8000 imaging system (AlphaInnotech, San Leandro, CA, USA). The gray values were analyzed by AlphaEaseFC 4.0.

### Bacterial Infection

The mouse model of bacterial sepsis was established as previously described (5, 42). *E. coli* (DH5α strain) was grown in Luria Broth (LB) media at 37°C overnight, and then reinoculated into fresh LB media and grown for 4 h at 37°C. The viable bacteria were collected by centrifugation at 2,600 × *g* for 10 min, washed with PBS, and then resuspended in appropriate volume of PBS. Bacterial density was measured by using an ultraviolet-visible spectrophotometer (NanoDrop2000, Thermo Scientific), and the corresponding colony-forming units (CFUs) were determined on LB agar plates. All mice were acclimated for 1 week, randomly divided into three groups, and intragastrically administered once with baicalin solution (100 or 200 mg/kg body weight) or vehicle (2% Tween-80 in PBS). Three hours later, viable *E. coli* cells (2 × 10^9^ CFU/mouse) in 0.5 ml of PBS were injected into the peritoneal cavity of each mouse. Mice were intragastrically administered once again with baicalin solution or vehicle 1 h after bacterial infection. Mouse survival was monitored every 6 h for five consecutive days. In another experiment, mice were treated similarly and were sacrificed at 4 and 8 h post bacterial infection. Their sera were collected, and serum IL-1β levels were measured by CBA mouse IL-1β Flex Set.

### Statistical Analysis

All experiments were performed three times independently, with one representative experiment shown. The data were expressed as mean ± SD and analyzed for statistical significance using GraphPad Prism 5.0 (GraphPad Software Inc., San Diego, CA, USA). One-way analysis of variance followed by Tukey *post hoc* test and unpaired Student’s *t*-test were used to analyze the statistical significance among multiple groups and between two groups, respectively. Kaplan–Meier survival curves were adopted for analysis of mouse survival, and the statistical difference between two groups was determined using the log-rank (Mantel–Cox) test. *P*-values < 0.05 were considered statistically significant.

## Results

### Baicalin Inhibits NLRP3 Inflammasome Activation in BMDMs

**Figure 1:**
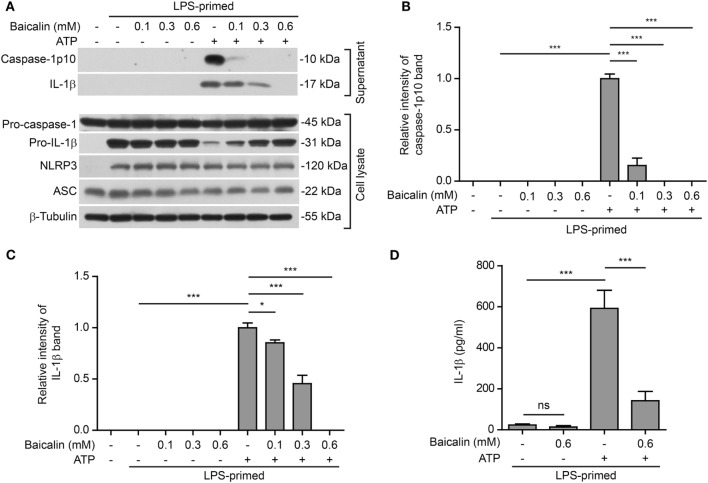
Baicalin suppressed adenosine triphosphate (ATP)-induced activation of NOD-like receptor (NLR) family, pyrin containing domain 3 (NLRP3) inflammasome. Bone marrow-derived macrophages were first primed with LPS (500 ng/ml) for 4 h and then pretreated with graded concentrations of baicalin for 1 h, followed by incubation with ATP (3 mM) for 30 min in the absence of LPS. **(A)** Western blotting was used to assess the expression levels of indicated proteins in the cell lysates and culture supernatants, respectively. β-Tubulin was used as a loading control for cell lysates. **(B,C)** Histograms showing the relative intensity of capase-1p10 **(B)** or interleukin (IL)-1β **(C)** bands in culture supernatants in **(A)**. The intensity of capase-1p10 or IL-1β bands in ATP group was set to 1.0. The intensity of the other groups was calculated relative to the ATP group. **(D)**. Cells were treated as in panel **(A)**. The levels of soluble IL-1β were detected by cytometric bead array assay in the culture supernatants. The experiments were performed three times independently, with one representative experiment shown. Data are shown as mean ± SD (*n* = 3). *P < 0.05; ***P < 0.001; ns, not significant.

NOD-like receptor (NLR) family, pyrin containing domain 3 inflammasome activation in macrophages needs two signals: PAMP (including LPS) priming (signal 1) and DAMP activation (signal 2) (3). We sought to explore whether baicalin could influence the activation of NLRP3 inflammasome in BMDMs. Cells were first primed with LPS (signal 1) and then pretreated with graded doses of baicalin before stimulation with NLRP3 activator ATP (signal 2). Western blotting was used to detect the two commonly used markers of inflammasome activation: the enzymatically active p10 subunit of caspase-1 (caspase-1p10) and mature IL-1β (17 kDa) in the culture supernatants. Consistent with previous reports (45), our data showed that both pro-IL-1β and NLRP3 proteins were highly induced by LPS, whereas pro-caspase-1 and ASC were constitutively expressed in macrophages irrespective of LPS priming (Figure 1A). Upon ATP treatment, both active caspase-1p10 (indicative of caspase-1 activation) and mature IL-1β were released into the culture supernatants. However, baicalin robustly suppressed the release of activated caspase-1p10 and mature IL-1β into the culture supernatant (Figures 1A–C). Notably, without ATP treatment, baicalin alone neither changed the constitutive expression nor the induced expression (by LPS) of the inflammasome related proteins (Figure 1A). Besides, bead-based immunoassay (CBA) was also used to detect mature IL-1β in culture supernatants. As expected, baicalin reduced ATP-induced secretion of mature IL-1β (Figure 1D), confirming the result of Western blot analysis of IL-1β (Figure 1A).

**Figure 2:**
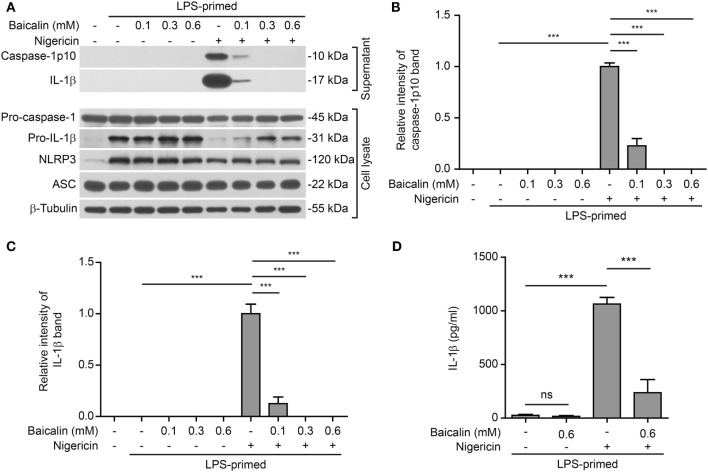
Baicalin attenuated nigericin-induced NOD-like receptor (NLR) family, pyrin containing domain 3 (NLRP3) inflammasome activation. Bone marrow-derived macrophages were first primed with LPS (500 ng/ml) for 4 h and then pretreated with indicated doses of baicalin for 1 h, followed by incubation with nigericin (10 μM) for 1 h without LPS. **(A)** Western blot analysis was used to assess the expression and secretion of indicated proteins in the cell lysates and culture supernatants, respectively. β-Tubulin was used as a loading control for cell lysates. **(B,C)** Histograms showing the relative intensity of capase-1p10 **(B)** or interleukin (IL)-1β **(C)** bands in culture supernatants in panel **(A)**. The intensity of capase-1p10 or IL-1β bands in nigericin group was set to 1.0. The intensity of the other groups was calculated relative to the nigericin group. **(D)** The levels of soluble IL-1β were detected by cytometric bead array assay in the culture supernatants. The experiments were performed three times independently, with one representative experiment shown. Data are shown as mean ± SD (*n* = 3). ***P < 0.001; ns, not significant.

Further, we tested whether baicalin could affect NLRP3 inflammasome activation by another NLRP3 activator nigericin (a microbial toxin derived from *Streptomyces hygroscopicus*, acting as a potassium ionophore). In line with the results derived from ATP stimulation, baicalin also markedly reduced caspase-1 activation and mature IL-1β secretion in a dose-dependent manner upon nigericin stimulation (Figures 2A–D), indicating the suppression of NLRP3 activation. Taken together, these results indicated that baicalin inhibited NLRP3 inflammasome activation in macrophages upon ATP or nigericin stimulation.

### Baicalin Inhibits ATP- or Nigericin-Induced Cell Death in LPS-Primed BMDMs

**Figure 3:**
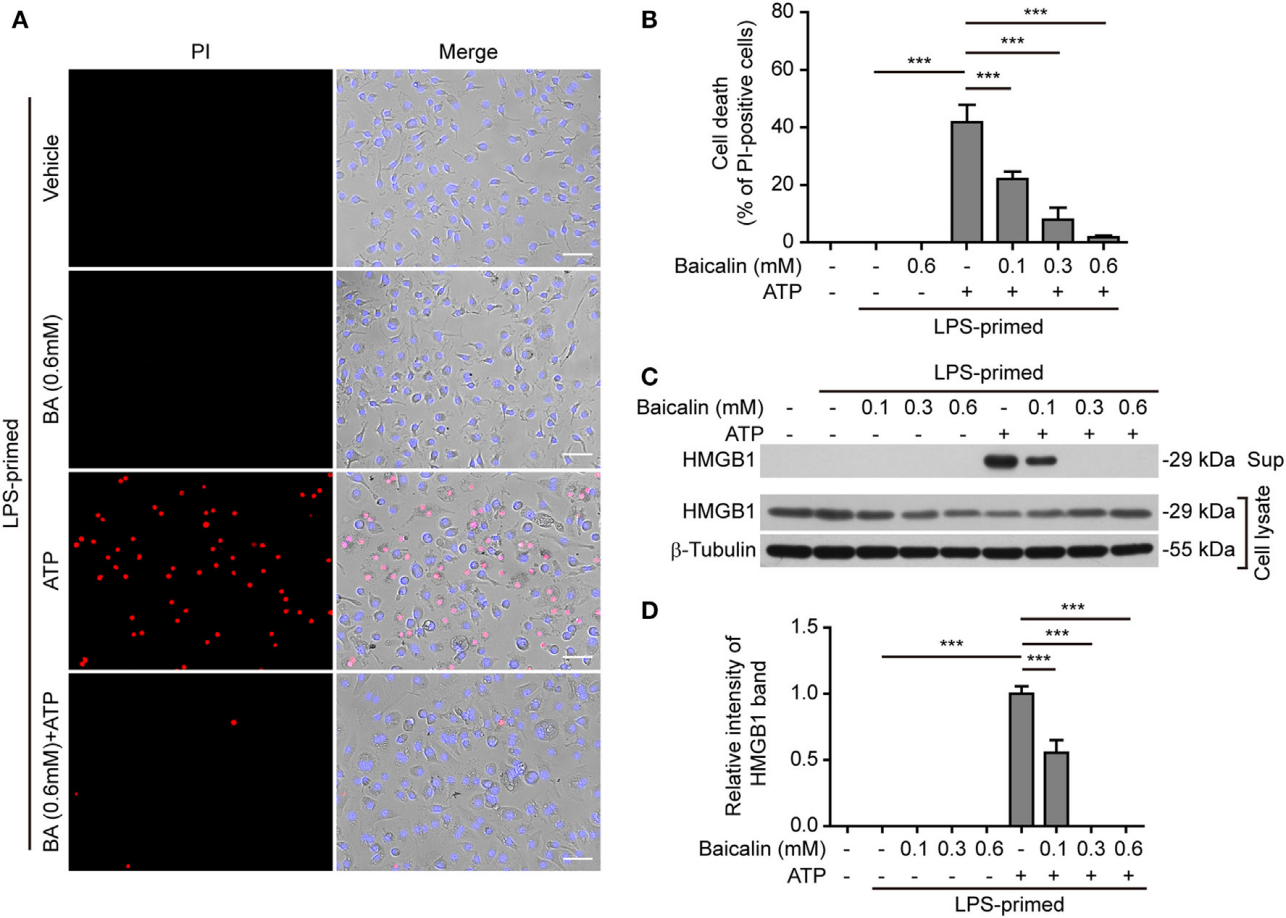
Baicalin inhibited adenosine triphosphate (ATP)-induced cell death in macrophages. Bone marrow-derived macrophages were treatment as in Figure 1A. **(A)** Cell death was measured by staining with propidium iodide (PI) (red, staining dead cells) and Hoechst 33342 (blue, staining all cells) together for 10 min. All images were captured by fluorescence microscopy and showed in merge with bright-field images. One set of representative images of three independent experiments is shown. Scale bars, 50 µm. **(B)** PI-positive cells in five randomly chosen fields with each containing ~100 cells were quantified. The percentage of cell death is defined as the ratio of PI-positive cells relative to all cells (revealed by Hoechst). Data are shown as mean ± SD (*n* = 5). **(C)** Cells were treated as in panel **(A)**. Western blotting was used to assess the expression levels of indicated proteins in the cell lysates and culture supernatants, respectively. β-Tubulin was adopted as a loading control for cell lysates. **(D)** Histograms showing the relative intensity of high-mobility group box-1 (HMGB1) band in culture supernatants in panel **(C)**. The intensity of HMGB1 band in ATP group was set to 1.0 while those of the other groups were calculated relative to the ATP group. The experiments were performed three times independently, with one representative experiment shown. Data are shown as mean ± SD (*n* = 3). ***P < 0.001; BA, baicalin; Sup, supernatant.

**Figure 4:**
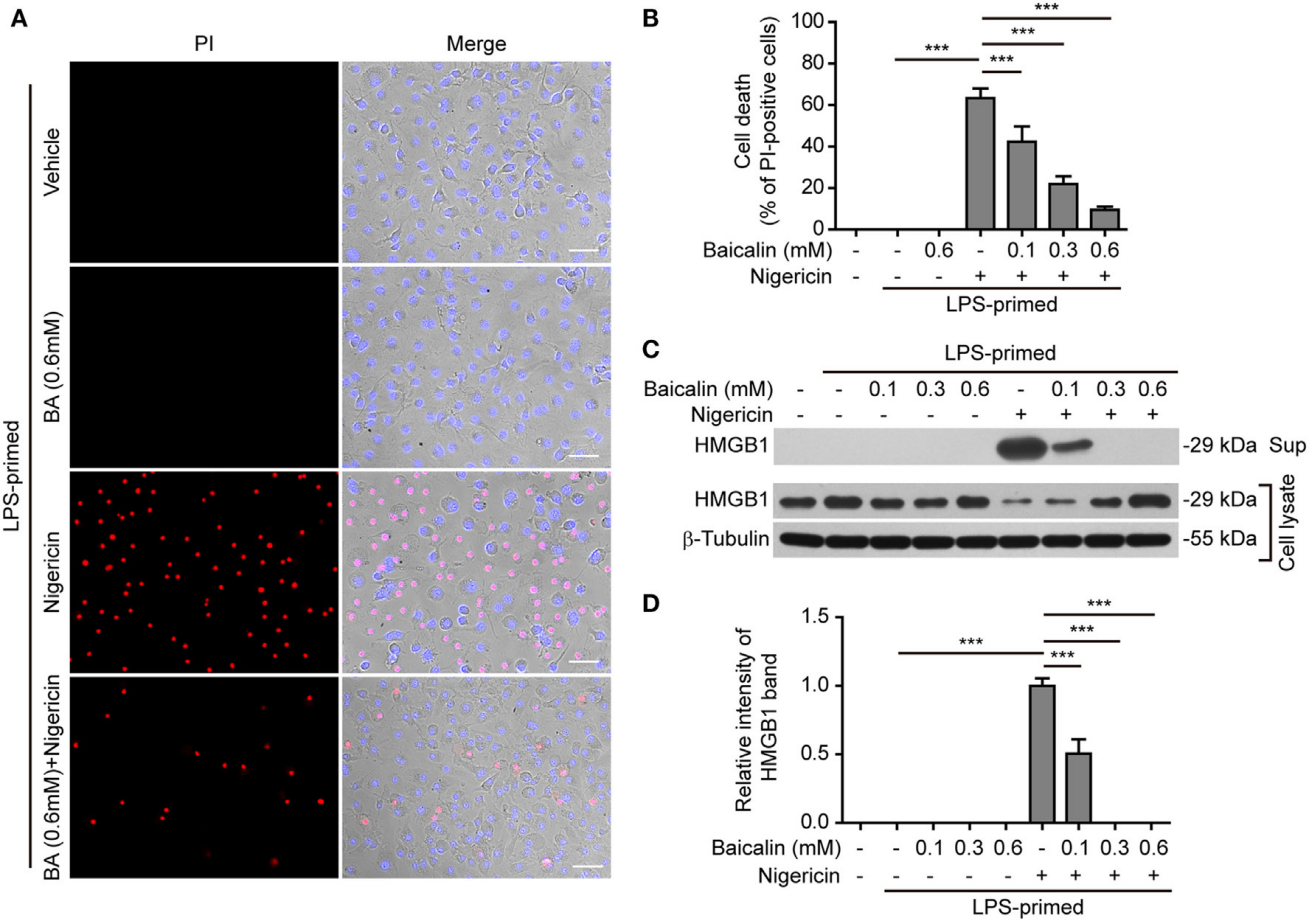
Baicalin inhibited nigericin-induced cell death in macrophages. Bone marrow-derived macrophages were treated as indicated in Figure 2A. **(A)** Cell death was measured by staining with propidium iodide (PI) (red, staining dead cells) and Hoechst 33342 (blue, staining all cells) together for 10 min. All the images were captured by fluorescence microscopy and showed in merge with bright-field images. One set of representative images of three independent experiments is shown. Scale bars, 50 µm. **(B)** PI-positive cells in five randomly chosen fields each containing ~100 cells were quantified. The percentage of cell death is defined as the ratio of PI-positive cells relative to all cells (revealed by Hoechst). Data are shown as mean ± SD (*n* = 5). **(C)** Cells were treated as in panel **(A)**. Western blotting was used to assess the expression levels of indicated proteins in the cell lysates and culture supernatants, respectively. β-Tubulin was used as a loading control for cell lysates. **(D)** Histograms showing the relative intensity of high-mobility group box-1 (HMGB1) band in the culture supernatants in panel **(C)** (*n* = 3). The intensity of HMGB1 band in nigericin group was set to 1.0, and those of the other groups were calculated relative to the nigericin group. The experiments were performed three times independently, with one representative experiment shown. Data are shown as mean ± SD (*n* = 3). ***P < 0.001; BA, baicalin; Sup, supernatant.

NOD-like receptor (NLR) family, pyrin containing domain 3 inflammasome triggering leads to the activation of caspase-1 rapidly culminating in an inflammatory form of cell death—pyroptosis (7, 8), which can be detected either by PI staining (40) or by measuring released cellular components including HMGB1 in the culture supernatants (46, 47). We thus explored whether baicalin inhibited ATP- or nigericin-induced cell death in BMDMs. LPS-primed cells were treated with ATP, and cell death was monitored by using fluorescence microscopy. As shown in Figure 3A, PI staining showed that cell death was rapidly induced by ATP. The proportion of ATP-induced cell death was ~45% (Figure 3B). Consistent with its effects on suppressing NLRP3 activation, baicalin markedly decreased the ratios of ATP-induced cell death in a dose-dependent manner (Figure 3B). Further supporting this, baicalin dose-dependently inhibited the release of HMGB1 into the culture supernatants (Figures 3C,D). Without ATP triggering, however, baicalin alone induced neither cell death nor HMGB1 release. Similarly, baicalin remarkably attenuated nigericin-induced cell death (Figures 4A,B) and the release of HMGB1 into the culture supernatants but left more HMGB1 in the cell lysates (Figures 4C,D). Taken together, these results indicated that baicalin suppressed ATP-or nigericin-induced NLRP3 inflammasome activation and cell death in macrophages.

### Baicalin Blocks ASC Speck Formation and Oligomerization upon NLRP3 Activation

**Figure 5:**
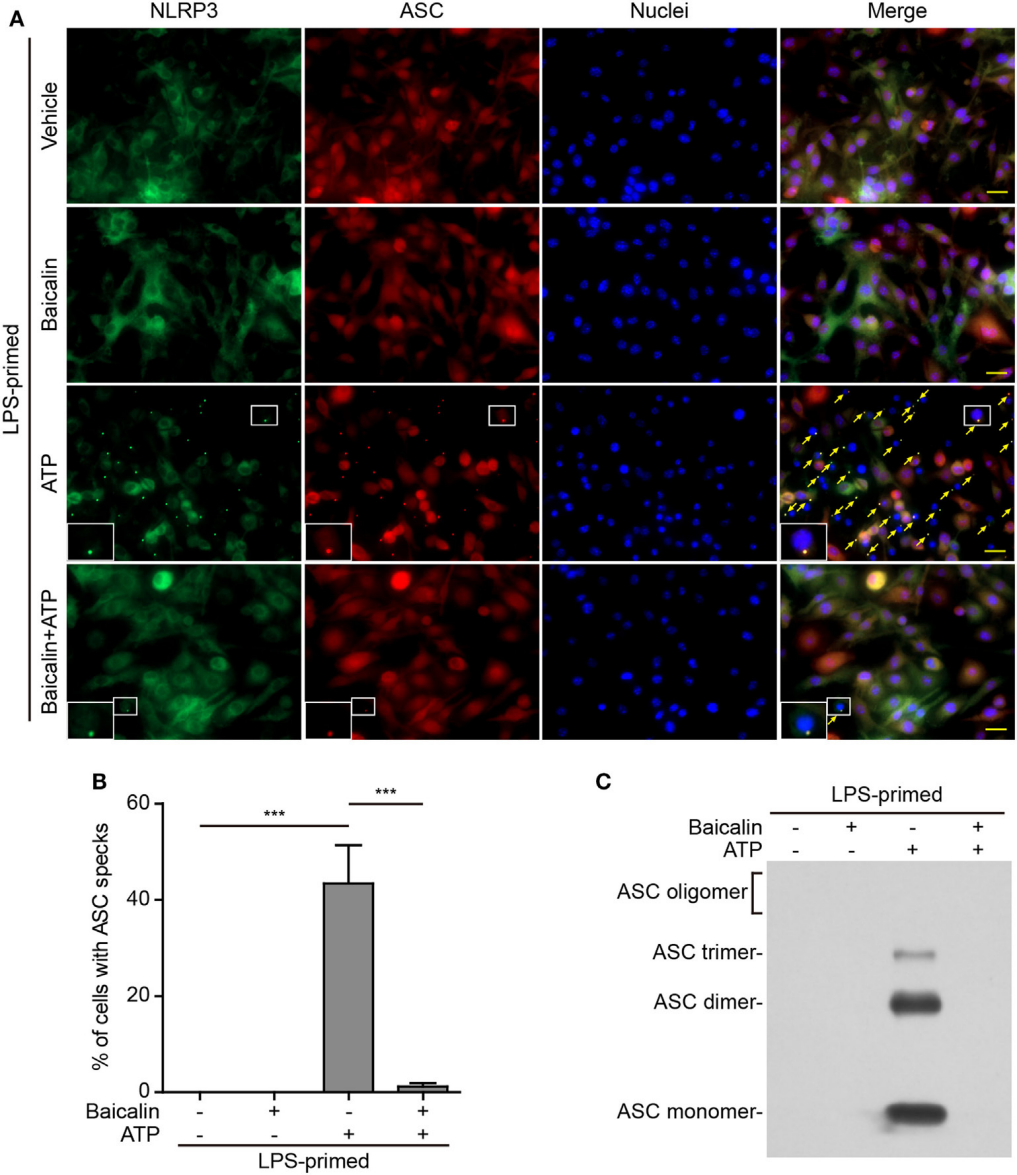
Baicalin blocked adenosine triphosphate (ATP)-induced ASC speck formation and oligomerization. Bone marrow-derived macrophages were primed with LPS (500 ng/ml) for 4 h, pretreated with baicalin (0.6 mM) for 1 h, and triggered with ATP (3 mM) for 30 min in the absence of LPS. **(A)** Representative immunofluorescence images showing ASC (red) and NOD-like receptor (NLR) family, pyrin containing domain 3 (NLRP3) (green) subcellular distributions. Nuclei (blue) were stained with Hoechst 33342. Merged images were also presented to show the co-localization of ASC specks and NOD-like receptor (NLR) family, pyrin containing domain 3 (NLRP3) dots in the cytoplasm. Yellow arrows indicate the ASC specks, and the enlarged insets show cells with an ASC speck. Scale bars, 20 µm. **(B)** Quantification of cells with ASC specks relative to the total number of cells in five randomly chosen fields with each containing ~50 cells. **(C)** Western blot analysis for ASC in Triton-X 100 insoluble pellets cross-linked with disuccinimidyl suberate. The experiments were performed three times independently, with one representative experiment shown. Data are shown as mean ± SD (*n* = 3). ***P < 0.001.

**Figure 6:**
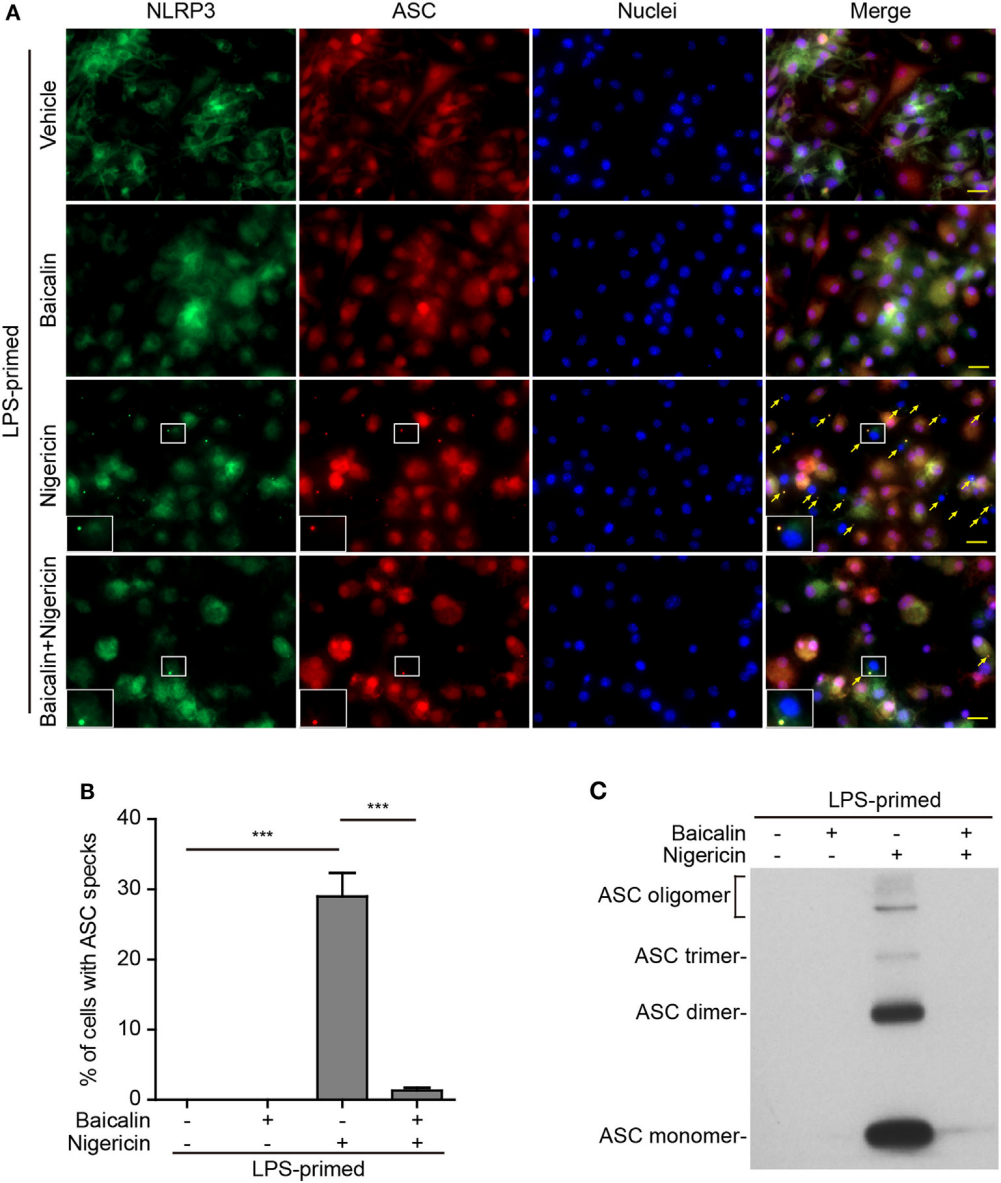
Baicalin suppressed nigericin-induced ASC speck formation and oligomerization. Bone marrow-derived macrophages were primed with LPS (500 ng/ml) for 4 h, pretreated with baicalin (0.6 mM) for 1 h, and triggered with nigericin (10 µM) for 1 h without LPS. **(A)** Representative immunofluorescence images showing ASC (red) and NOD-like receptor (NLR) family, pyrin containing domain 3 (NLRP3) (green) subcellular distribution. Nuclei (blue) were stained with Hoechst 33342. Merged images are also presented to show the co-localization of ASC specks and NLRP3 dots in the cytoplasm. Yellow arrows indicate ASC specks, and the enlarged cell showed with an ASC speck. Scale bars, 20 µm. **(B)** Percentages of cells containing ASC specks relative to the total number of cells in five randomly chosen fields with each containing ~50 cells. **(C)** Western blot analysis for ASC in Triton-X 100 insoluble pellets cross-linked with disuccinimidyl suberate. The experiments were performed three times independently, with one representative experiment shown. Data are shown as mean ± SD (*n* = 3). ***P < 0.001.

Upon activation, NLRP3 recruits its downstream adaptor ASC to form large multi-protein complexes, which can be revealed as specks by either immunofluorescence microscopy or be evidenced as ASC oligomers by Western blotting after chemical cross-linking. We firstly analyzed the formation of ASC specks by fluorescence microscopy. Consistent with the Western blotting results (Figures 1A and 2A), immunofluorescence analysis demonstrated that NLRP3 was weakly detected in unstimulated macrophages but was highly expressed in LPS-primed cells, whereas ASC expression was unaffected by LPS priming (Figure S1 in Supplementary Material). Without stimulation of NLRP3, ASC and NLRP3 were diffusely distributed in cells regardless of baicalin and/or LPS treatment, whereas ATP or nigericin treatment led to ASC speck formation in ~40 or ~30% of the cells, respectively (Figures 5A,B and 6A,B). Notably, ASC specks were highly co-localized with NLRP3 dots in the cytoplasm (Figures 5A and 6A), indicating the recruitment of ASC and formation of NLRP3 inflammasomes in these macrophages. Remarkably, baicalin treatment before ATP or nigericin stimulation drastically decreased the percentages of cells containing ASC specks to 3 or 1%, respectively (Figures 5A,B and 6A,B), suggesting that baicalin could suppress the activation of NLRP3 inflammasome by blocking ASC speck formation.

As ASC specks formed after NLRP3 activation are insoluble in PBS containing 0.5% Triton-X 100 thus being collected as pellets and the ASC proteins within the pellets can be cross-linked by disuccinimidyl suberate to form oligomers (43), we examined the effect of baicalin on such ASC oligomerization by Western blot analysis. In LPS-primed macrophages, different ASC complexes (dimers, trimers, and higher oligomers) were observed in the samples treated with ATP or nigericin, whereas no ASC was detectable in the cells treated with vehicle or baicalin alone indicating that ASC was soluble in these cell lysates. Similar to the ASC speck assay, ATP or nigericin-induced ASC oligomerization was almost completely blocked by baicalin (Figures 5C and 6C). Together, these data further confirmed that baicalin treatment inhibited NLRP3 inflammasome activation through blocking ASC recruitment into the NLRP3 inflammasome.

### Baicalin-Mediated Inhibition of NLRP3 Inflammasome Activation and IL-1β Release Is Reversed by the PKA Pathway Inhibitors

**Figure 7:**
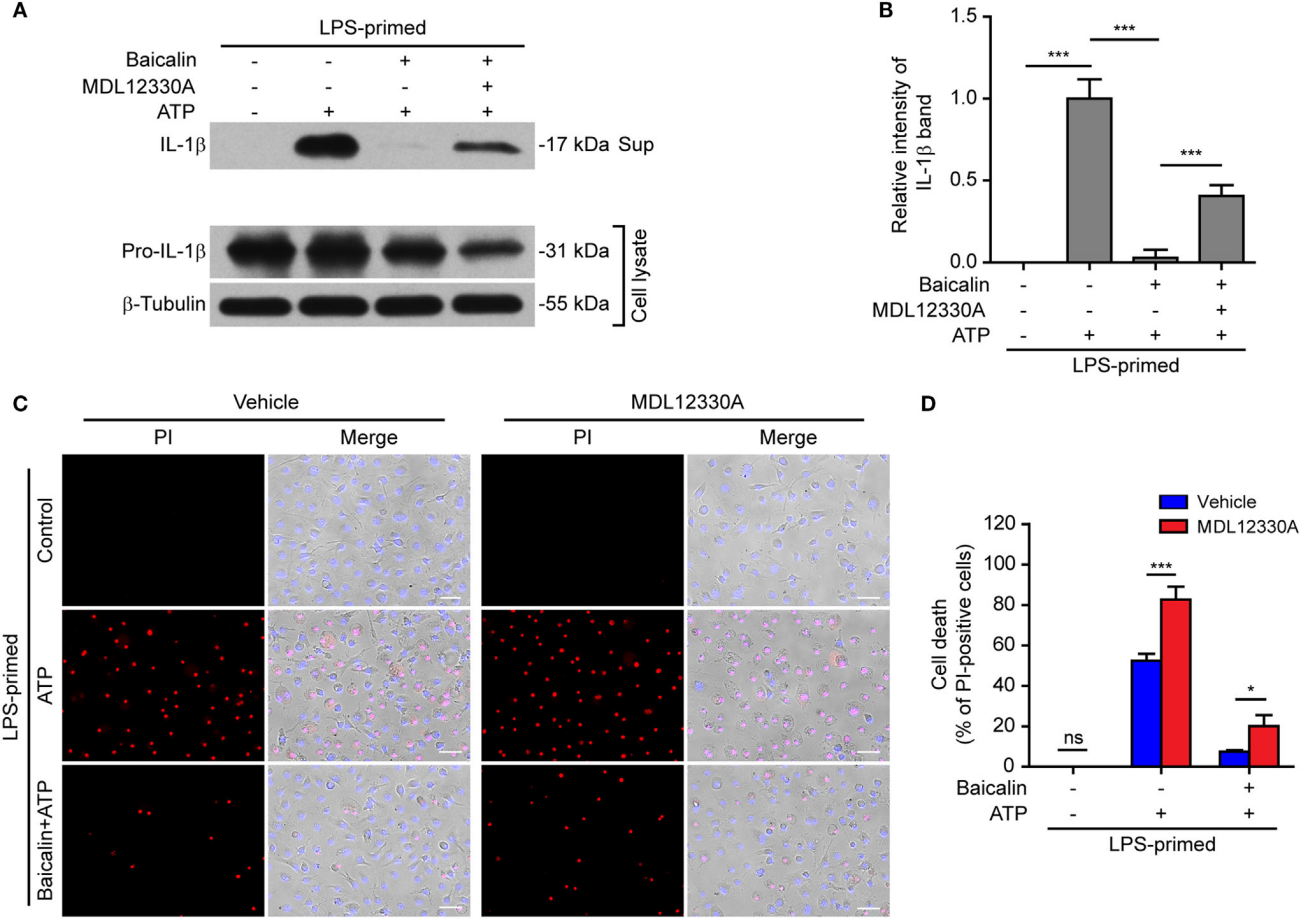
Baicalin inhibited NOD-like receptor (NLR) family, pyrin containing domain 3 (NLRP3) inflammasome activation depending on the adenyl cyclase activity. Bone marrow-derived macrophages were primed with LPS (500 ng/ml) for 4 h, pretreated with baicalin (0.3 mM) for 30 min, and then incubated with adenyl cyclase inhibitor MDL12330A (10 µM) for 30 min, followed by co-treatment with adenosine triphosphate (ATP) (3 mM) for 30 min. **(A)** Western blot analysis was used to detect the expression and secretion of interleukin (IL)-1β in the cell lysates and culture supernatants, respectively. β-Tubulin was used as a loading control for cell lysates. **(B)**. Histograms showing the relative intensity of mature IL-1β band in panel **(A)** (*n* = 3). The intensity of IL-1β band in ATP group was set to 1.0 while those of the other groups were calculated relative to the ATP group. **(C)** Cell death was measured by staining with propidium iodide (PI) (red, staining dead cells) and Hoechst 33342 (blue, staining all nuclei) together for 10 min. All images were captured by fluorescence microscopy and showed in merge with bright-field images. One set of representative images of three independent experiments is shown. Scale bars, 50 µm. **(D)** PI-positive cells in five randomly chosen fields each containing ~100 cells were quantified. The percentage of cell death is defined as the ratio of PI-positive cells relative to all cells (revealed by Hoechst). Data are shown as mean ± SD (*n* = 5). *P < 0.05; ***P < 0.001; ns, not significant; Sup, supernatant.

**Figure 8:**
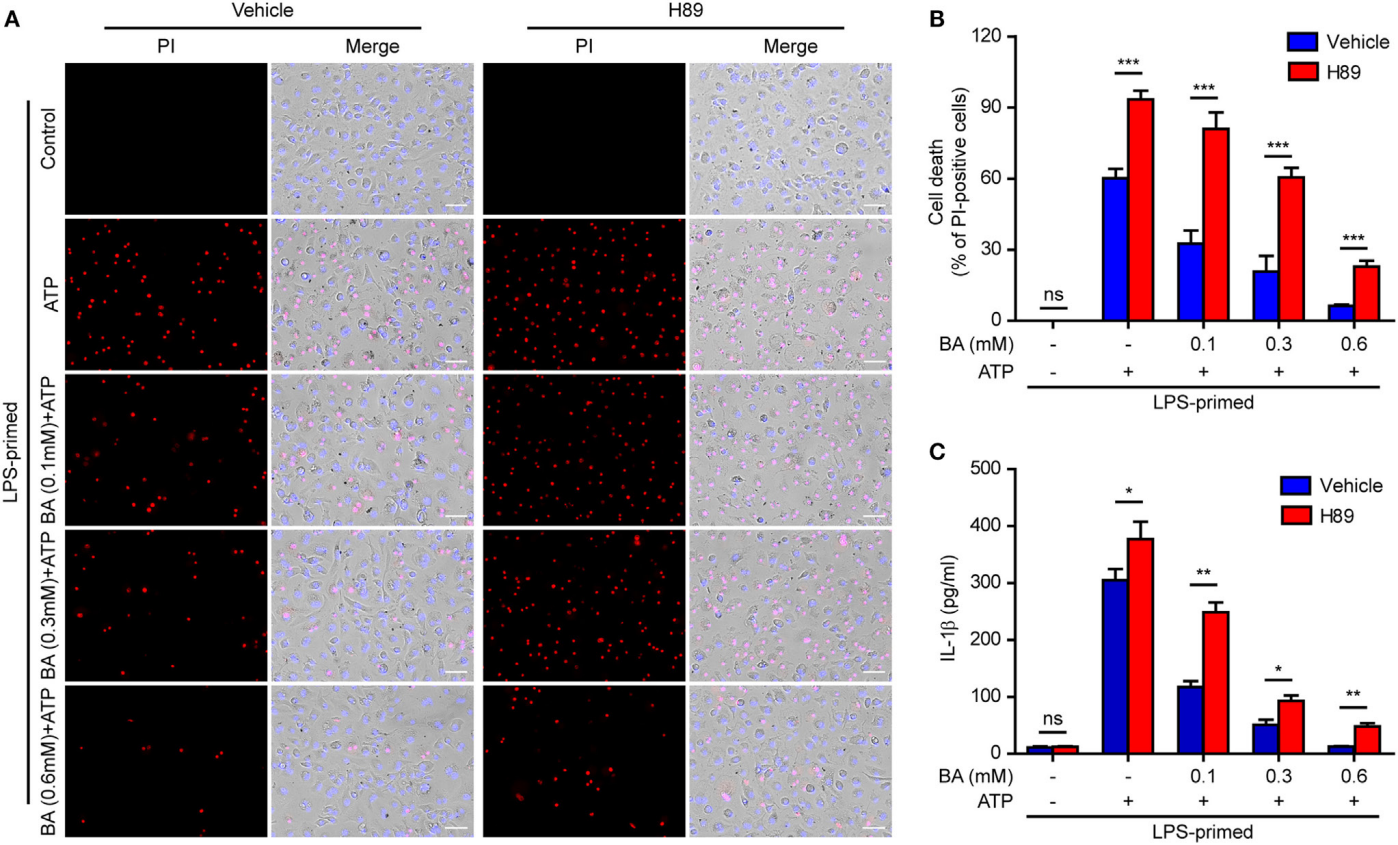
Baicalin inhibited cell death in macrophages depending on protein kinase A (PKA) signaling. LPS-primed bone marrow-derived macrophages were pretreated with the PKA inhibitor H89 (20 µM) for 30 min and then incubated with graded doses of baicalin for 1 h, followed by stimulated with adenosine triphosphate (ATP) (3 mM) for 30 min. **(A)** Cell death was assay by propidium iodide (PI) (red) and Hoechst 33342 (blue) co-staining for 10 min. The images were captured by fluorescence microscopy. One set of representative images of three independent experiments is shown. Scale bars, 50 µm. **(B)** PI-positive cells in five randomly chosen fields each containing ~100 cells were quantified. The percentage of cell death is defined as the ratio of PI-positive cells relative to all cells (revealed by Hoechst). Data are shown as mean ± SD (*n* = 5). **(C)** Cells were treated as in panel **(A)**. The levels of soluble interleukin (IL)-1β in culture supernatants were measured by cytometric bead array assay. Data are shown as mean ± SD (*n* = 3). *P < 0.05; **P < 0.01; ***P < 0.001; ns, not significant; BA, baicalin.

In light of previous findings that the PKA negatively regulates NLRP3 activation (37, 38) and that baicalin may increase the PKA activity (48), we next explored whether baicalin suppressed the NLRP3 inflammasome activation by upregulating PKA signaling in macrophages. First, the adenyl cyclase inhibitor MDL12330A was used to block cyclic AMP (cAMP) production thus attenuating the PKA activity. As shown in Figures 7A,B, the effect of baicalin on suppressing mature IL-1β (17 kDa) release upon ATP stimulation was partly antagonized by MDL12330A treatment. Furthermore, MDL12330A not only increased ATP-induced cell death but also counteracted the effect of baicalin on suppressing the cell death (Figures 7C,D). The PKA inhibitor H89 had similar effects as MDL12330A did, counteracting the inhibitory effect of baicalin on ATP-induced cell death (Figures 8A,B) and IL-1β release (Figure 8C). Together, these results suggested that baicalin suppressed NLRP3 inflammasome activation at least in part through the PKA signaling.

### Baicalin-Mediated Suppression of ASC Speck formation Is Counteracted by Blocking PKA Signaling

**Figure 9:**
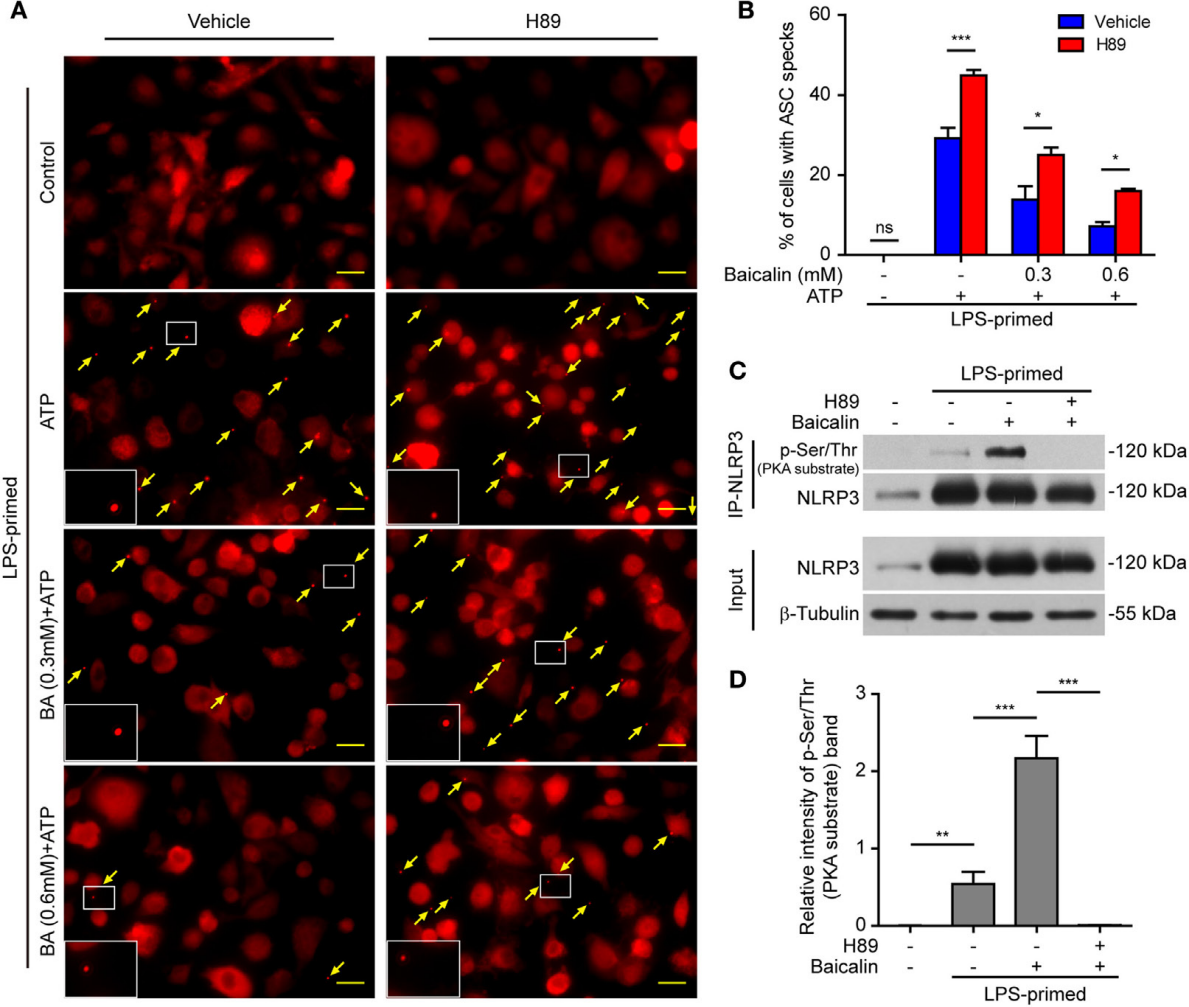
Baicalin inhibited ASC speck formation by upregulating protein kinase A (PKA) signaling. Bone marrow-derived macrophages (BMDMs) were treated as described in Figure 8. **(A)** Representative immunofluorescence images showing ASC subcellular distribution. Yellow arrows indicate ASC specks. The enlarged insets showed cells with an ASC speck. Scale bars, 20 µm. **(B)** The ASC speck formation was quantified by the cells with ASC specks relative to the total cells from five random fields each containing ~50 cells. **(C)** LPS-primed BMDMs were incubated with the PKA inhibitor H89 (20 µM) for 30 min and then treated with baicalin (0.6 mM) for 1 h without adenosine triphosphate (ATP) treatment. NOD-like receptor (NLR) family, pyrin containing domain 3 (NLRP3) immunoprecipitation was analyzed for phosphorylation on PKA-specific sites (p-Ser/Thr PKA substrate). **(D)** The intensity of p-Ser/Thr phosphorylation on PKA-specific sites of NLRP3 was quantified relative to total NLRP3 in panel **(C)**. *P < 0.05; **P < 0.01; ***P < 0.001; ns, not significant; BA, baicalin.

As baicalin could attenuate ASC speck formation upon NLRP3 activation, we next explore whether the suppression of ASC speck formation by baicalin could also be reversed by blocking the PKA activity. The PKA inhibitor H89 increased the ASC speck formation induced by ATP, and it could partly reversed the inhibitory effect of baicalin on ATP-induced ASC speck formation (Figures 9A,B), further confirming that PKA signaling had been involved in the action of baicalin on suppressing NLRP3 inflammasome activation.

Therefore, we next explored whether baicalin could enhance the Ser/Thr phosphorylation of NLRP3 on PKA-specific sites, which has been reported to prevent NLRP3 inflammasome activation in macrophages (37, 38). To this end, NLRP3 was immunoprecipitated, and its phosphorylation on Ser/Thr residues of PKA substrate motifs was evaluated by Western blotting. Indeed, the PKA-specific phosphorylation on Ser/Thr residues of NLRP3 was greatly increased by baicalin pretreatment. The PKA inhibitor H89 blocked such phosphorylation, further verifying that baicalin-enhanced NLRP3 phosphorylation was mediated by PKA signaling (Figures 9C,D). Altogether, these results suggested that baicalin inhibited NLRP3 inflammasome activation at least partly through augmenting PKA signaling.

### Baicalin Treatment Increases Mouse Survival in Bacterial Sepsis

**Figure 10:**
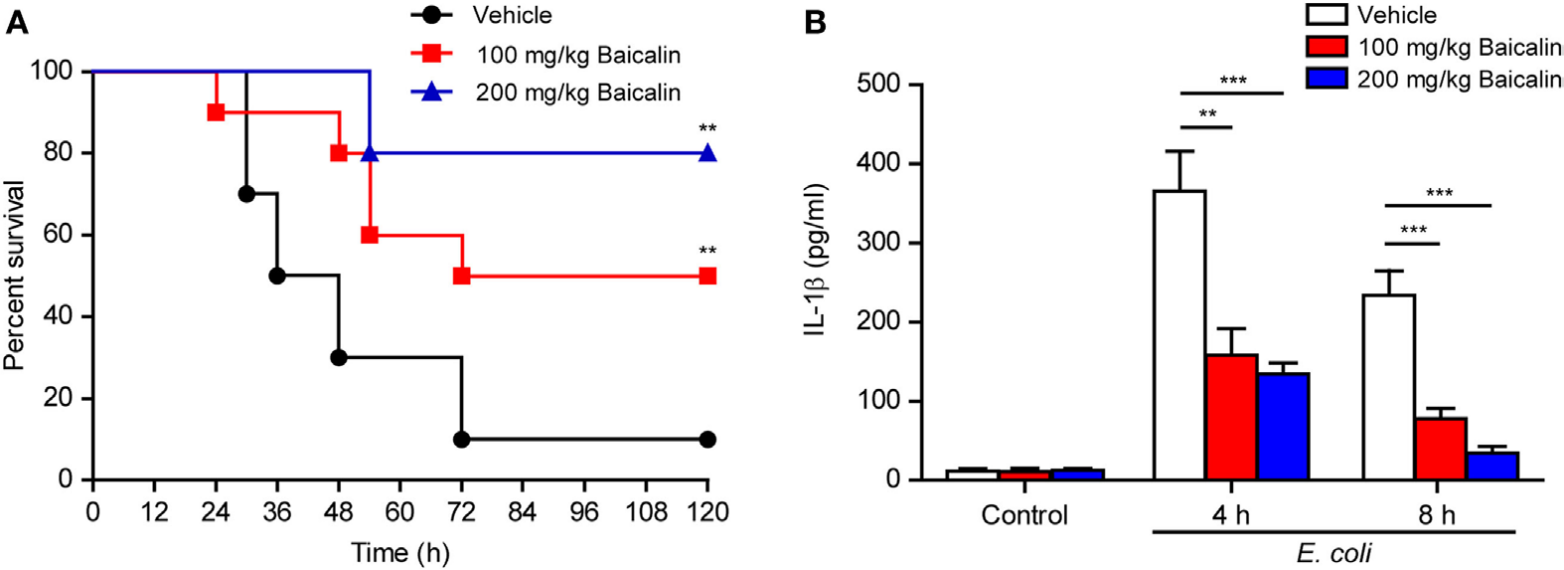
Baicalin administration prolonged mouse survival in bacterial sepsis. **(A)** C57BL/6 mice were administered (intragastrically) with baicalin (100 or 200 mg/kg body weight) or vehicle (2% Tween-80 in PBS) once 3 h before peritoneal injection with viable *Escherichia coli* (2 × 10^9^ CFU/mouse). One hour after the bacterial injection, mice were intragastrically administered with baicalin or vehicle once again. Mouse survival was monitored every 6 h for five consecutive days. Kaplan–Meier survival curves were used to analyze the data (10 mice per group). The significance was evaluated by the log-rank (Mantel–Cox) test. **(B)** Mice were treated as in panel **(A)**. The serum levels of interleukin (IL)-1β at 4 and 8 h post bacterial infection were measured by cytometric bead array assay (five mice per group). **P < 0.01; ***P < 0.001.

As ATP-induced NLRP3 inflammasome activation has critical roles in bacterial sepsis (5), we explored whether baicalin could ameliorate sepsis in a mouse model of bacterial infection in the peritoneal cavity. Mice were administered once intragastrically with baicalin solution (100 or 200 mg/kg body weight) or vehicle 3 h before intraperitoneal injection of viable *E. coli* (2 × 10^9^ CFU/mouse). One hour after the injection of bacteria, the mice were administered once again with baicalin or vehicle. Only 10% of mice survived the period of observation (120 h) in the vehicle group, whereas baicalin administration significantly increased their survival rates when compared with the vehicle group (Figure 10A). Moreover, baicalin treatment significantly decreased IL-1β levels in the sera of bacterial infected mice as compared with vehicle (Figure 10B), indicating that baicalin suppressed systemic inflammation in the mice, which was consistent with the *in vitro* studies showing baicalin’s inhibitory effects on NLRP3 activation. Therefore, this result highlights the potential application of baicalin in the treatment of inflammatory diseases including bacterial sepsis.

## Discussion

In this study, we found that baicalin markedly inhibited NLRP3 inflammasome activation in LPS-primed macrophages upon ATP or nigericin triggering, thereby blocking the activation of caspase-1 and the release of mature IL-1β and HMGB1. This inhibitory action of baicalin is in part mediated by augmentation of PKA signaling as the adenyl cyclase inhibitor MDL12330A or PKA inhibitor H89 could partly reverse such an effect. Indeed, baicalin enhanced PKA-mediated phosphorylation of NLRP3, thereby reducing NLRP3’s capacity to recruit the adaptor ASC and the formation of ASC oligomerization. In line with the finding that baicalin inhibited NLRP3 inflammasome activation *in vitro*, baicalin administration *in vivo* significantly improved the survival of mice in bacterial sepsis.

During bacterial infection, ATP can be released either from the host’s monocytes/macrophages or from bacterial cells (5, 49, 50). By acting on the purinergic P2X7 receptor on the plasma membrane, extracellular ATP can induce the efflux of K^+^, thus triggering the NLRP3 inflammasome leading to the activation of caspase-1 (51–53). The activated caspase-1 further leads to pyroptosis and release of inflammatory cytokines including IL-1β and HMGB1 (7, 8, 13, 46, 47), which greatly exacerbate the inflammatory responses during bacterial infections and even leading to organ damage and septic shock (54–56). We showed in this study that baicalin markedly inhibited NLRP3 inflammasome activation in murine macrophages upon ATP stimulation, suggesting that baicalin may attenuate the severity of bacterial sepsis. Consistent with this notion, we found that baicalin could significantly improve the survival of mice in a model of bacterial sepsis. This result is in line with several previous studies showing that baicalin can prolong the survival of mice in both CLP-induced sepsis and LPS-induced endotoxemia (28–30). Consistent with our results, these previous reports showed that baicalin did reduce the levels of HMGB1 in septic mice and suppress the release of IL-1β and HMGB1 from macrophages (29), even though not clearly pointing to NLRP3 inflammasome activation. In support of our findings, two separate studies provided evidence that baicalin could suppress the NLRP3 inflammasome pathway in piglet mononuclear phagocytes in response to LPS stimulation (36) or *Haemophilus parasuis* infection (35). Those previous studies and our data indicated that baicalin robustly inhibited NLRP3 inflammasome activation to attenuate inflammatory responses during infections such as bacterial sepsis.

More recently, there is evidence indicating that PKA signaling negatively regulates NLRP3 activation. For example, bile acid induces an increase in intracellular cAMP levels through acting on its receptor TGR5, leading to PKA activation and PKA-mediated phosphorylation of NLRP3, which suppresses NLRP3 inflammasome activation in macrophages (37). It has also been shown that prostaglandin E2 can inhibit NLRP3 inflammasome activation by inducing PKA signaling (38). In addition, dopamine negatively regulates NLRP3 inflammasome *via* a cAMP-dependent manner (57). Consistent with these studies, we in this study provided evidence that baicalin suppressed NLRP3 activation by modulating PKA activity. Baicalin greatly increased PKA-mediated Ser/Thr phosphorylation of NLRP3 and prevented ATP-induced formation of ASC specks, but these effects of baicalin could be counteracted by blocking the PKA signaling pathway with its specific inhibitors. This indicated that baicalin prevented NLRP3 inflammasome assembly by enhancing the NLRP3 phosphorylation on PKA-specific sites. In further support of this, PKA inhibitors also counteracted baicalin’s effects on suppressing ATP-induced caspase-1p10 and IL-1β release. In addition, ATP-induced NLRP3 inflammasome activation was further enhanced by PKA inhibitor, which is in line with previous studies (37, 38). Collectively, these data demonstrated that baicalin can suppress NLRP3 inflammasome activation by, at least partly, modulating PKA signaling.

Furthermore, blocking cAMP formation by inhibiting the adenyl cyclase (AC) activity also significantly reversed baicalin-induced suppression of NLRP3 activation, highlighting the possibility that baicalin may modulate upstream components of PKA signaling. It was likely that baicalin might have increased cAMP levels by modulating AC activity, although we had not assayed the cAMP levels. In support of this notion, a previous study showed that baicalin administration could increase cAMP levels *in vivo* (48). However, the intracellular levels of cAMP are also regulated by its degradation enzyme phosphodiesterases (PDEs) (58). A large panel of PDEs has been shown to regulate cAMP levels thus modulating the PKA activity and NLRP3 activation. For example, the PED4 inhibitor Rolipram has been shown to inhibit NLRP3 inflammasome activation in submandibular gland cells (59). Although we had shown that AC inhibitor could reverse baicalin-mediated suppression of NLRP3, our data does not exclude the possibility that baicalin may regulate PKA signaling by affecting the activity of PDEs. Recently, it has been shown that the phosphorylation of NLRP3 on Ser 3 (in mouse, which corresponds Ser 5 in human) has a critical role in modulating NLRP3 activation and that the protein phosphatase PP2A is involved in dephosphorylation and activation of NLRP3 inflammasome (60). As the motif surrounding Ser 3 (MTS*) is different from the PKA substrate motif [(K/R)(K/R)XS*/T*], it is still unknown whether Ser/Thr phosphorylation of NLRP3 on PKA-specific sites is regulated by PP2A. Besides, although H89 did inhibit the action of PKA on NLRP3 as revealed by abrogating the phosphorylation on PKA-specific sites, it should be noted that H89 may have other action targets beyond PKA (61). Therefore, further investigation is warranted to elucidate the precise mechanism by which baicalin modulates the cAMP/PKA signaling pathway.

As aforementioned, NLRP3 inflammasome activation requires two signals: the priming (first signal by LPS, etc.) and triggering (second signal by ATP or nigericin, etc.) (3, 62). Baicalin has been shown to exhibit anti-inflammatory activity by suppressing the NF-κB pathway, thus suppressing the expression of inflammatory cytokines and NLRP3 inflammasome components under various circumstances (31–36). It is worth noting that in those studies such inhibitory effects of baicalin appear to be attributed to the first signal (priming) of NLRP3 inflammasome (35, 36), thus leading to the downregulation of NLRP3 activation since the full activation of this inflammasome is relying on a marked upregulation of this key protein. However, it was unclear whether baicalin could inhibit NLRP3 inflammasome activation at the triggering step (second signal). In this study, we found that baicalin had minimal effect on the expression of NLRP3 inflammasome components, including the constitutive expression of ASC and pro-caspase-1 as well as induced expression of NLRP3 and pro-IL-1β in LPS-primed macrophages. Instead, baicalin could block NLRP3 inflammasome activation upon ATP or nigericin triggering, indicative of its action on the second signal for this inflammasome. Such inhibitory effects of baicalin are likely mediated by augmenting the PKA signaling leading to increased phosphorylation of NLRP3 on PKA-specific sites, which in turn prevents the adaptor ASC recruitment into the inflammasome. Therefore, apart from its inhibitory effects on NLRP3 transcription as revealed by previous studies (36, 37), baicalin can also inhibit NLRP3 inflammasome activation in macrophages upon ATP or nigericin triggering.

Adenosine triphosphate and nigericin have been widely used as activators to stimulate NLRP3 inflammasome activation in LPS-primed macrophages (6, 37, 38, 44), accompanied by caspase-1-dependent cell death, which is regarded as pyroptotic cell death (2, 3). Supporting this, one recent study elegantly showed that gasdermin D, NLRP3, and caspase-1 are all needed for nigericin to induce cell death and IL-1β secretion in LPS-primed RAW 264.7 cells expressing ASC, indicating that nigericin does induce pyroptosis in LPS-primed macrophages (11). However, it has been found that a diverse array of NLRP3 activators, including ATP and nigericin, induced necrosis in human THP-1 cells irrespective of LPS stimulation (63). One possible explanation for this discrepancy is that the cell death types induced by NLRP3 activators may be dependent on experimental settings (such as incubation time and stimulator concentrations). For example, in most of studies (6, 37, 38, 44), ATP (2–5 mM) and nigericin (10 µM) were incubated for 0.5–1 h to activate the NLRP3 inflammasome, respectively. But longer nigericin incubation time (4–6 h) and higher ATP concentrations (≥30 mM for 4–6 h) had been shown to trigger necrosis (63). In this study, we used nigericin stimulation for 1 h and found that baicalin significantly inhibited nigericin-induced inflammasome activation and cell death in LPS-primed macrophages, suggesting that baicalin could inhibit NLRP3-mediated pyroptosis. Similarly, as LPS plus ATP treatment induced caspase-1 activation and IL-1β release, it was likely that the accompanied cell death was pyroptosis. Further studies are warranted to uncover whether baicalin inhibits ATP- or nigericin-induced necrosis under the condition without LPS priming.

In conclusion, we found that baicalin robustly inhibited ATP or nigericin-induced NLRP3 inflammasome activation in mouse macrophages, at least partly *via* augmenting PKA signaling. Furthermore, we also showed that baicalin administration significantly prolonged mouse survival in a model of bacterial sepsis, suggestive of suppression of systemic inflammation. Although future research is warranted to unravel the precise mechanism of baicalin’s action on the PKA pathway and investigations in human macrophages may provide more informative data, our studies highlight that baicalin can be used for the treatment of NLRP3-related inflammatory diseases including bacterial sepsis.

## Ethics Statement

All animal experiments were performed according to the guidelines for the care and use of animals approved by the Committee on the Ethics of Animal Experiments of Jinan University.

## Author Contributions

C-GL, LY, F-YM, and Y-YJ performed *in vitro* studies; L-HX, Z-JS, and Q-BZ conducted animal studies; C-GL, LY, and Z-JS analyzed the data; D-YO and X-HH supervised the study; X-HH, D-YO, and C-GL wrote the paper.

## Conflict of Interest Statement

The authors declare that the research was conducted in the absence of any commercial or financial relationships that could be construed as a potential conflict of interest.
