# Supplementary material for: Baicalin Inhibits NOD-Like Receptor Family, Pyrin Containing Domain 3 Inflammasome Activation in Murine Macrophages by Augmenting Protein Kinase A Signaling
Source: Front Immunol. 2017 Oct 27;8:1409. doi: 10.3389/fimmu.2017.01409 (PMC5674921; doi:10.3389/fimmu.2017.01409)
Supplement: Supplementary file 1 [file presentation_1.pdf]

Supplementary figure

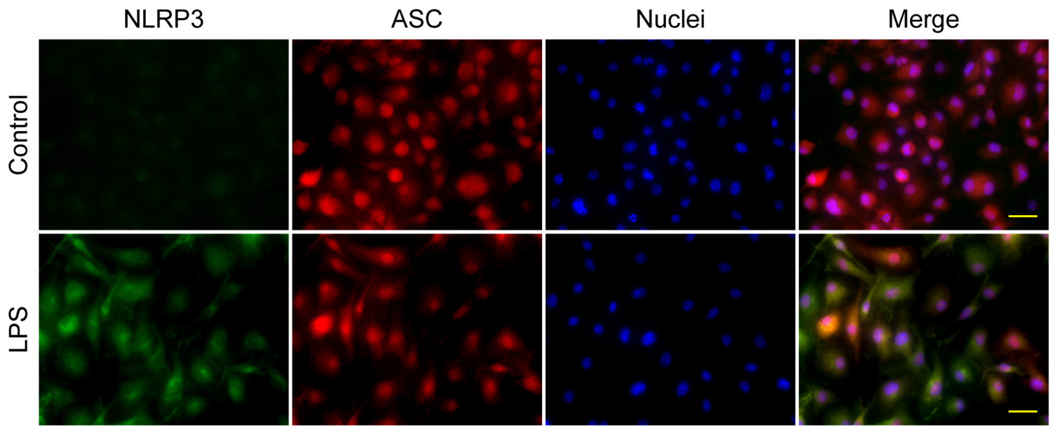

Supplementary Figure 1 | NLRP3 protein was highly expressed in LPS-primed macrophages. Mouse bone marrow-derived macrophages (BMDMs) were primed with or without 500 ng/ml LPS stimulation for 4 h. Cells were fixed, stained and observed by using fluorescence microscopy. Representative immunofluorescent images were captured, showing NLRP3 (green) and ASC (red) subcellular distribution. Nuclei (blue) were stained with Hoechst 33342. Scale bars, 20 μm.
